# Incidence of Venous Thromboembolism and Its Associated Risk Factors in Newly Diagnosed Multiple Myeloma Patients in the Klang Valley, Malaysia

**DOI:** 10.3390/jcm14030759

**Published:** 2025-01-24

**Authors:** Nurul Izzati Abdul Aziz, Sivakumar Palaniappan, Nor Saaidah Kamal Rodin, Guang Yong Chong, Tan Tsen Chuen Jerome, Azimatun Noor Aizuddin, Nor Rafeah Tumian

**Affiliations:** 1 Department of Medicine, Faculty of Medicine, Universiti Kebangsaan Malaysia, Jalan Yaacob Latif, Bandar Tun Razak, Kuala Lumpur 56000, Malaysia; izzati.aziz6818@gmail.com (N.I.A.A.); siva_redknapp@hotmail.com (S.P.); craycgy@gmail.com (G.Y.C.); 2 Hospital Canselor Tuanku Muhriz (HCTM), Universiti Kebangsaan Malaysia, Jalan Yaacob Latif, Bandar Tun Razak, Kuala Lumpur 56000, Malaysia; saaidah@hctm.ukm.edu.my; 3 Hematology Department, Hospital Ampang, Jalan Mewah Utara, Ampang 68000, Selangor, Malaysia; romeje@gmail.com; 4 Department of Public Health Medicine, Faculty of Medicine, Universiti Kebangsaan Malaysia, Jalan Yaacob Latif, Bandar Tun Razak, Kuala Lumpur 56000, Malaysia; azimatunnoor@hctm.ukm.edu.my

**Keywords:** myeloma, thrombosis, incidence, risk factors, risk assessment

## Abstract

**Background:** Venous thromboembolism (VTE) is a potentially severe medical problem among multiple myeloma (MM) patients, with evolving treatment regimens potentially increasing the thrombotic risk. Data on VTE incidence and risk factors in multiethnic Malaysian MM patients are limited. This study aimed to assess VTE incidence and risk factors in newly diagnosed MM (NDMM) patients at two tertiary centres in Klang Valley, Malaysia. **Methods:** This retrospective cohort study included NDMM patients, aged ≥18, diagnosed between January 2015 and December 2022 at Hospital Canselor Tuanku Muhriz and Hospital Ampang. Patient demographics, clinical characteristics, MM therapies, and thromboprophylaxis data were analysed. VTE is defined as deep vein thrombosis (DVT) or pulmonary embolism (PE), confirmed by imaging. **Results:** Among the 216 NDMM patients (mean age: 62.4 ± 10.6 years), 22 (10.2%) developed VTE (15 DVT, five PE, and two both). The median time from MM diagnosis to VTE was 3.5 months (IQR 5.3). A univariate analysis identified the female sex, an ECOG performance status ≥ 2, diabetes mellitus, a recent orthopaedic surgery (<6 months), a SAVED score ≥ 2, and an IMPEDE-VTE score > 3 as significant risk factors. In the multivariable logistic regression, the female sex (aOR 8.56, 95% CI: 1.95–37.48), an ECOG status ≥ 2 (aOR 12.74, 95% CI: 3.37–48.17), and a recent orthopaedic surgery (aOR 21.79, 95% CI: 3.10–153.38) were the independent risk factors of VTE among NDMM patients. **Conclusions:** VTE incidence in our NDMM cohort was 10.2%. Independent risk factors included the female sex, a poor performance status, and a recent orthopaedic surgery. Individualised thromboprophylaxis strategies are crucial, warranting further real-world studies to optimise anticoagulation regimens.

## 1. Introduction

Multiple myeloma (MM), a hematologic malignancy characterised by the clonal proliferation of plasma cells, accounts for approximately 1% of all cancers and 10% of hematologic malignancies. In Malaysia, MM is a relatively rare malignancy, with the Global Cancer Observatory (GLOBOCAN) reporting 370 new cases in 2022, representing 0.72% of all cancer diagnoses [1]. Venous thromboembolism (VTE), encompassing deep vein thrombosis (DVT) and pulmonary embolism (PE), is a significant cause of morbidity and mortality in cancer patients, including MM. The incidence of VTE has been estimated to be more than 10% during the course of the disease [2]. A Korean cohort study showed that VTE incidence was 120.4 per 1000 person-years within the first six months post-diagnosis [3]. In a community-based study by Ramasamy et al., the cumulative incidence was 13.1% at 12 months [4]. To date, there are limited data on VTE incidence and its associated risk factors among MM patients in Malaysia. This highlights the need for region-specific, real-world data to improve clinical decision-making and guideline development.

MM is associated with an increased risk of thrombotic events largely contributed by disease-specific mechanisms, patient comorbidities, and treatment-related factors. Among the patient-related factors, a history of prior VTE was found to be linked to a heightened risk in newly diagnosed multiple myeloma (NDMM) patients [5,6]. Several researchers found that other important contributing patient factors were an age of 65 years and older and comorbidities, such as congestive heart failure and hypertension [7,8]. The hypercoagulable state induced by MM is attributed to elevated immunoglobulin levels, the procoagulant activity of monoclonal proteins, and the presence of inflammatory cytokines, which collectively exacerbate thrombotic risks [9]. The therapeutic landscape for MM has evolved significantly with the introduction of novel agents, such as immunomodulatory drugs (IMiDs), proteasome inhibitors (PIs), and monoclonal antibodies. Current treatment protocols often incorporate these agents into triplet or quadruplet regimens to maximise the treatment response, followed by autologous stem cell transplantation (ASCT) for eligible patients and maintenance therapy using IMiDs or PIs [10]. However, the use of IMiDs, particularly in combination with chemotherapy and high-dose corticosteroids, has been associated with an increased incidence of thromboembolic complications [11,12,13]. The findings of these studies have improved our awareness regarding VTE in MM patients, especially during treatment with IMiDs. A randomised trial conducted by the Eastern Cooperative Oncology Group demonstrated that among 102 NDMM patients, those treated with thalidomide plus dexamethasone experienced a VTE rate of 17%, compared to 3% in patients receiving dexamethasone alone [14]. Barrett et al. reported a VTE incidence of 9.6% in a cohort of 332 NDMM patients in Ireland, with those who developed VTE having an associated mortality odds ratio of 3.3 compared to age-matched MM controls [15]. However, Li et al. found an IMiD-related VTE incidence of 6.1% among NDMM patients in China. Lower VTE rates have been documented with lenalidomide maintenance therapy following ASCT, showing an incidence of 6% over a median follow-up of 45 months [16].

The SAVED, IMPEDE-VTE, and PRISM scores are a few well-validated risk assessment models (RAMs) that have been developed and utilised in clinical practice to predict VTE risks in MM patients [5,6,17]. These models take into account various patient-specific factors, including age, sex, comorbidities, disease characteristics, and treatment regimens, to stratify patients into different risk categories. The SAVED score is specifically designed for MM patients receiving IMiDs and has been validated across multiple cohorts [6]. It incorporates five clinical variables: prior surgery, Asian race, history of VTE, age ≥ 80 years, and dexamethasone dosage. This model has shown significant predictive ability for the VTE risk in patients initiating IMiD therapy, with high-risk patients (SAVED score ≥ 2) exhibiting a markedly higher incidence of VTE compared to low-risk patients (SAVED score < 2) [6]. Similarly, the IMPEDE-VTE score offers a comprehensive assessment that includes broader patient- and treatment-related factors. It categorises patients into three risk groups based on their scores: low-risk (≤3), intermediate-risk (4–7), and high-risk (≥8) [5]. The cumulative incidence of VTE at six months was significantly higher in the high-risk group than in the low-risk group. Recognising the elevated risk of VTE, particularly within the first six months following an MM diagnosis, the 2022 National Comprehensive Cancer Network (NCCN) guidelines recommend thromboprophylaxis based on calculated scores from either the SAVED or IMPEDE-VTE score, provided there are no contraindications to anticoagulation or antiplatelet-therapy [10]. For patients scoring ≤ 3 on the IMPEDE score or <2 on the SAVED score, aspirin (81–325 mg daily) is advised. Conversely, higher-risk patients may be prescribed enoxaparin (40 mg/day subcutaneously), warfarin (target INR 2.0–3.0), fondaparinux (2.5 mg/day subcutaneously), or direct oral anticoagulants (DOACs) such as rivaroxaban or apixaban [10].

In the real world, implementing thromboprophylaxis in patients with MM remains a complex challenge, influenced by several factors. Increased bleeding risks associated with cancer treatments, severe anaemia at diagnosis, and frailty in elderly patients complicate the decision-making process for clinicians. A study by Bradbury et al. highlighted that VTE events continue to occur frequently throughout the MM treatment course, adversely affecting the overall prognosis even when primary thromboprophylaxis is employed [18]. This highlights the persistent risk of VTE despite preventive measures.

To the best of our knowledge, no published local data exist on the incidence of VTE and its associated risk factors among NDMM patients in Klang Valley, Malaysia. This study aimed to determine the incidence of VTE in NDMM patients treated at Hospital Canselor Tuanku Muhriz (HCTM) and Hospital Ampang. Specifically, this study sought to (i) identify risk factors associated with VTE, including patient-, disease-, and treatment-related factors, and (ii) evaluate bleeding events, including major bleeding and clinically relevant non-major bleeding (CRNMB), as well as the causes of in-hospital mortality among MM patients with VTE. This study highlights the burden of VTE in NDMM patients in the Klang Valley, providing valuable insights to guide future research. Furthermore, it highlights the need for clinicians to adopt current thromboprophylaxis guidelines to improve VTE-related outcomes in NDMM patients at our centres.

## 2. Materials and Methods

### 2.1. Study Design and Population

This retrospective cohort study involved NDMM patients diagnosed and receiving treatment at the Clinical Haematology Unit and Myeloma Clinic, HCTM and Department of Haematology, Hospital Ampang, Malaysia. Both are tertiary-level hospitals that provide haematology services in Kuala Lumpur, the capital of Malaysia. Consecutive NDMM patients aged 18 years and older who received treatment for at least one year between January 2015 and December 2022 were screened for this study. Patients with known coagulation or thrombotic disorders, such as Factor V Leiden or antiphospholipid syndrome, were excluded, as well as individuals with a prior history of malignancy, known cases of atrial fibrillation, valvular heart disease, and those who experienced VTE after an MM diagnosis but before the initiation of chemotherapy. Convenience sampling was performed. The study protocol received an approval from the Research Ethics Committee of Universiti Kebangsaan Malaysia (FF-2023-124) and the Medical Research and Ethics Committee (MREC) of the Malaysian Ministry of Health (MOH) (NMRR ID-23-00962-XG2). Given the retrospective nature of this study, informed consent was waived. However, the patients’ confidentiality was strictly maintained by de-identifying the data.

### 2.2. Data Collection Process

#### 2.2.1. Data Source

All the NDMM patients from January 2015 to December 2022 were identified using discharge codes from the 10th revision of the International Classification of Diseases (ICD), specifically codes C90 for multiple myeloma, I82 for deep vein thrombosis, and I26 for pulmonary embolism, and screened for their eligibility in this study. Patient lists were retrieved from the International Centre for Casemix and Clinical Coding for HCTM and the multiple myeloma database in the Haematology Department at Hospital Ampang, Selangor, Malaysia. Notably, this study had no duplicated data between the two patient registries. The data collection spanned from the time of multiple myeloma diagnosis until either death, loss to follow-up, or 31 August 2024 (end of the study period).

#### 2.2.2. Clinical and Laboratory Evaluation

Detailed demographic data were collected, including the age, gender and self-reported ethnicity around the time of MM diagnosis. The body mass index (BMI) was calculated using height and weight measurements within one month of diagnosis. The Eastern Cooperative Oncology Group (ECOG) performance status of each patient was graded from 0 to 5. Additionally, comorbidities such as diabetes mellitus (DM), chronic kidney disease (CKD), hypertension, dyslipidemia, ischemic heart disease (IHD), stroke, and any orthopaedic surgeries performed within six months of the MM diagnosis were reviewed.

Information on disease characteristics was extracted from the clinical case notes and the Integrated Laboratory Information Management System (ILMS). The data within the first two weeks of diagnosis included the date of diagnosis and the type and subtype of myeloma. Baseline laboratory values such as haemoglobin (Hb), serum calcium, M-protein levels (including serum and urine paraprotein), serum-free light-chain levels (FLC), β_2_-microglobulin levels at diagnosis, serum lactate dehydrogenase (LDH) levels, cytogenetic abnormalities, and staging based on the Durie–Salmon (DS) and Revised International Staging System (R-ISS) were recorded. Treatment data according to the respective institutional chemotherapy protocols within one year from the diagnosis were documented, including details on radiotherapy, central venous catheterisation (CVC) for chemotherapy administration, prescriptions for erythropoietin-stimulating agents, and information regarding transplants as a consolidation therapy. Clinical and laboratory data at the time of MM diagnosis were used to calculate the SAVED and IMPEDE-VTE scoring systems, and patients were further categorised into risk categories.

In this study, VTE events encompassed deep vein thrombosis (DVT), pulmonary embolism (PE), or venous thrombosis at other sites, including catheter-related thrombosis. Information on VTE events (date of diagnosis, site of VTE, type and duration of anticoagulation therapy, prior thromboprophylaxis measures, bleeding events, and all-cause in-hospital mortality following VTE events) was obtained from the medical records and electronic data system. According to the International Society on Thrombosis and Haemostasis (ISTH), major bleeding is defined by meeting at least one of the following three criteria: bleeding that is either fatal or symptomatic in a critical area or organ, leads to a decrease in Hb of 2 g/dL or more, or requires the transfusion of two or more units of red blood cells or whole blood [19]. Clinically relevant non-major bleeding (CRNMB) was defined as any sign or symptom of haemorrhage that does not fit the criteria for the ISTH definition of major bleeding requiring medical professional attention, hospitalisation, or evaluation in atrial fibrillation studies or ISTH major bleeding in non-surgical patients [20].

#### 2.2.3. Operational Definition

Deep vein thrombosis (DVT): the inability to fully compress the venous lumen with the pressure of the ultrasound probe during ultrasound (USG), combined with Doppler to help identify the deep veins and assess the presence of intraluminal thrombus, confirmed the diagnosis of DVT [21]. Pulmonary embolism (PE): any evidence of an intraluminal filling defect in a lobar or main pulmonary artery using computed tomography with pulmonary angiography (CTPA) or an intraluminal filling defect in a segmental pulmonary artery and moderate or high pretest clinical probability score fulfilled the diagnosis of PE [21].

### 2.3. Statistical Analysis

The statistical analysis was conducted using the Statistical Package for Social Sciences (SPSS) Version 29.0 software. Continuous variables were reported as the mean with standard deviation (SD) for normally distributed data or median with interquartile range (IQR) for non-normally distributed data. Categorical variables were expressed as counts with percentages. Chi-square or Fisher’s exact tests were used to compare categorical variables. For continuous variables, the t-test was applied to those that followed a normal distribution, while the Mann–Whitney test was performed for those that did not. Univariate and multivariate regression analyses were performed to identify risk factors associated with VTE following the diagnosis of MM, with results presented as odds ratios (OR) and 95% confidence intervals (CI). All variables deemed significant in the univariate analysis were included in the multivariate analysis. A *p*-value of less than 0.05 was considered statistically significant.

## 3. Results

### 3.1. Study Population and Disease Characteristics

**Figure 1:**
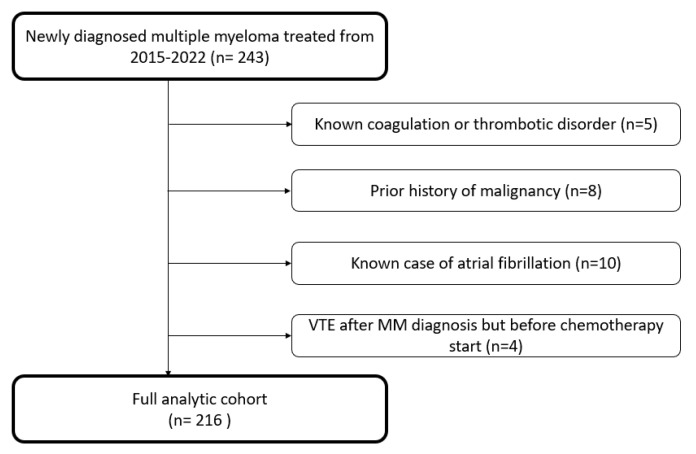
Overall study flowchart.

A total of 243 patients were initially screened for eligibility in the study. As illustrated in Figure 1, 216 NDMM patients were included in the final analysis. The median follow-up was 62 (IQR 30) months.

The demographic characteristics of MM patients are illustrated in Table 1. The cohort’s mean age at MM diagnosis was 62.4 (10.6) years. The gender distribution was almost similar. The ethnic composition mirrored Malaysia’s demographics, with a significant proportion of Malay patients, followed by Chinese (26.9%), Indian (11.1%), and Orang Asli, Iban, and Kadazan (1.9%). Most patients (82.4%) had a good performance status, classified as ECOG PS 0-1. The most common comorbidities among our NDMM patients included DM (71.3%), CKD (60.2%), and hypertension (41.7%). Ten patients (4.5%) underwent orthopaedic surgeries due to pathological fractures: eight had total hip replacements for femoral fractures, one received a vertebroplasty for a painful spinal compression fracture, and another underwent plating for a humeral fracture.

Of the patients, 130 (60.9%) had IgG type, with 81 (37.5%) Ig G kappa and 49 (22.7%) Ig G lambda. Most patients were classified under the DS and R-ISS as having advanced multiple myeloma. At the time of MM diagnosis, the mean Hb level was 8.31 (1.2) g/dL, the median serum β2-microglobulin level was 6.5 (IQR 5.7) mg/L, and the median serum calcium and albumin were 3.30 (IQR 0.6) mmol/L and 29.0 (IQR 3.0) g/L, respectively. Treatment was administered at the discretion of the attending haematologists at the respective centres. A total of 11 patients (5.0%) received radiotherapy concurrently with chemotherapy: 3 for spinal cord compression due to pathological fractures, 4 for plasmacytoma on the scalp, and 4 for painful pathological fractures. Central venous catheterisation (CVC) was performed in 98 patients (45.4%). One hundred and thirty-seven patients (63.4%) received an erythropoietin-stimulating agent (ESA) to treat anaemia. Additionally, 85 patients (39.4%) underwent autologous SCT as a consolidation therapy. The definitive treatment for MM within one year of diagnosis is listed in Table 1. During the induction therapy phase at our centres, a minimum triplet regimen was administered, with 88 patients (40.7%) receiving the VTD combination (bortezomib, thalidomide, and dexamethasone. Seventy patients (32.4%) were treated with the VRD regimen (bortezomib, lenalidomide, and dexamethasone). The remaining 58 patients (26.9%) received the VCD regimen (bortezomib, cyclophosphamide, and dexamethasone). None of our cohorts was prescribed newer agents, such as pomalidomide, carfilzomib, or daratumumab, as first-line therapy, given the cost issue.

### 3.2. Comparison of Socio-Demographic and Clinical Characteristics Between Patients with and Without VTE

Patients who developed VTE had significantly lower Hb and serum albumin levels and higher levels of serum β_2_-microglobulin, calcium, and lactate dehydrogenase (LDH) at diagnosis compared to those in the non-VTE group. Regarding the host factors, sex, ECOG performance status, diabetes mellitus, and recent orthopaedic surgery within six months of myeloma diagnosis were significantly associated with VTE (Table 1). There was no statistically significant association between the MM treatment and VTE.

### 3.3. VTE Events

#### 3.3.1. VTE Thromboprophylaxis

**Figure 2:**
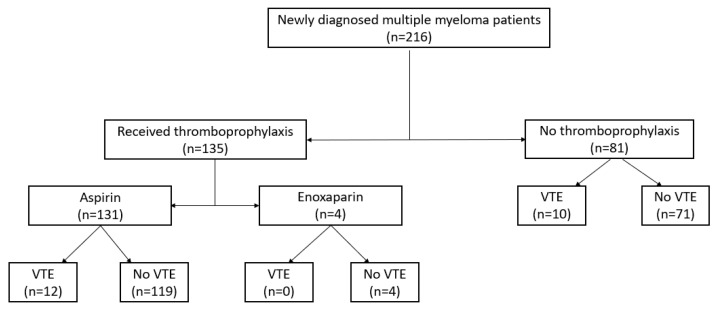
Thromboprophylaxis and VTE events in myeloma patients.

A total of 135 patients (62.5%) received thromboprophylaxis. Most received aspirin as thromboprophylaxis, in which 12 out of 131 developed VTE. In contrast, 10 of the 81 patients who did not receive thromboprophylaxis experienced VTE. Four patients (1.9%) were given prophylaxis enoxaparin at 0.5 mg/kg once daily, and none developed VTE (Figure 2).

We also analysed the association between SAVED and IMPEDE-VTE scores and VTE events. In our study, there was a significant association between SAVED and IMPEDE-VTE scores and VTE (Table 2).

#### 3.3.2. VTE Management

During the study period, 22 patients developed VTE, resulting in an incidence of 10.2%. The median time from MM diagnosis to VTE events was 3.5 months (IQR 5.3). Most of the patients experienced VTE within six months of their myeloma diagnosis. All patients exhibited symptoms during the VTE events. Details of the VTE events are shown in Table 3. None of our patients had catheter-related thrombosis or thrombosis recurrence after the completion of the anticoagulation therapies was reported.

In total, 16 patients (72.7%) completed the anticoagulant treatment, with a median treatment duration of 6.0 months (IQR 4.0). Due to disease progression, one patient continued receiving a prophylactic dose of enoxaparin after one year of treatment.

### 3.4. Risk Factors for VTE

A logistic regression analysis was performed to exclude the influence of confounding factors and identify factors independently related to VTE occurrence in Table 4. In this analysis, the following variables were shown to be independent risk factors for VTE in our MM patients: female gender (aOR 8.56 [95% CI: 1.95, 37.48], *p* = 0.004), ECOG performance status ≥ 2 (aOR 12.74 [95% CI: 3.37, 48.17], *p* < 0.001), and recent orthopaedic surgery within six months of myeloma diagnosis (aOR 21.79 [95% CI: 3.10, 153.38], *p* = 0.002).

### 3.5. Bleeding Events and All Causes of Mortality

Among the twenty-two myeloma patients with VTE, two patients experienced significant bleeding following the initiation of rivaroxaban. Both patients had upper gastrointestinal bleeding secondary to Forrest Ib and Forrest IIa ulcers, respectively, that required repeated blood transfusions and esophagogastroduodenoscopy (OGDS). The overall in-hospital mortality in MM patients with VTE was 5 out of 22 patients (22.7%). The causes of in-hospital mortality in our cohort were severe sepsis due to severe pneumonia and MM progression. There was no mortality directly associated with a thrombosis event.

## 4. Discussion

Information on VTE among MM patients in the Southeast Asian region remains limited. In our NDMM cohort, the incidence of VTE was approximately 10.2%, with most events occurring within the first six months following diagnosis. This rate was higher than those reported in other Asian countries [22,23,24]. In Western countries, the cumulative incidences were reported to be between 8–15% [2,18,25]. The increased incidence of VTE in our study may be attributed to differences in the study design, patient demographics, and disease characteristics. Our sample primarily comprised patients from two tertiary centres in the Klang Valley, where advanced-stage myeloma cases are referred to within or from nearby states. During induction therapy, our patients were seen at daycare once or twice a week. The frequent visits and clinical assessment may have contributed to the number of VTE events detected in our population during the induction periods, and a low threshold for imaging enabled early VTE confirmation if there was clinical suspicion. Additionally, convenience sampling may have led to a higher representation of patients with VTE.

DVT, particularly affecting the femoral vein, was our cohort’s predominant site of VTE. The median time to VTE occurrence was 3.5 months (IQR 5.3) from the diagnosis of MM, which was in agreement with findings that highlight an elevated risk of VTE within the first-year post-diagnosis [25,26]. The ROADMAP-MM-CAT study similarly reported a symptomatic VTE incidence of 10.4% during follow-up, with over half of these events taking place within three months after treatment initiation [27]. This increased risk can be attributed to multiple factors, including advanced disease stages, hypercoagulability associated with a heightened tumour burden, and active myeloma [9,28].

The NCCN guidelines recommend a risk-based approach for VTE prophylaxis in patients with MM, suggesting that low-risk patients should receive aspirin. In contrast, high-risk patients should be prescribed more potent anticoagulants such as enoxaparin, warfarin, fondaparinux, or DOACs, namely, rivaroxaban and apixaban [10]. In our cohort, 62.5% of the patients received thromboprophylaxis, indicating a growing awareness of VTE prevention strategies in managing MM. Interestingly, the incidence of VTE was lower among patients on thromboprophylaxis (9.1% with aspirin and none with enoxaparin) than those without prophylaxis (12.3%). However, the differences highlighted the controversy of primary thromboprophylaxis with aspirin and VTE events protection compared to LMWH and DOAC. A study by Frenzel et al. emphasised that aspirin should no longer prescribed as thromboprophylaxis in MM [29]. Despite efforts to minimise information bias by verifying available imaging in our computerised data, the small sample size and potential selection bias limit our ability to draw definitive and generalisable conclusions from these findings. Although current guidelines for primary thromboprophylaxis in MM patients offer a valuable framework, it is essential to thoroughly assess adherence to these guidelines and patient compliance. Future research should prioritise optimising physician prescribing practices for prophylactic strategies, enhancing patient adherence, and the effectiveness of these interventions to improve outcomes in this vulnerable population

The findings of our study revealed several patient factors as independent risk factors for VTE in NDMM patients. The prevalence of comorbidities such as diabetes mellitus and chronic kidney disease may contribute to an increased risk of VTE, as these conditions are known to exacerbate thrombotic tendencies. However, our study revealed no statistically significant association within the cohort. We found that the female sex is associated with an eight-fold increased risk of VTE in patients with NDMM. This observation is similar to the research conducted by Awada et al., which reported a three-fold higher VTE rate among female patients during the peri-transplant period [30]. The increased risk in our cohort may be attributed to a higher prevalence of overweight and obese individuals (BMI ≥ 25.0 kg/m^2^) at 49% and older age (over 60) at 60% among female patients, many of whom also had multiple comorbidities. These factors can lead to immobility, resulting in venous stasis and consequently elevating the risk of VTE.

Performance status is a critical prognostic factor influencing treatment options following a myeloma diagnosis. The Eastern Cooperative Oncology Group (ECOG) performance status measures a patient’s functional ability and mobility. Reduced mobility can exacerbate blood stasis in the veins, potentially damaging the endothelial lining and promoting clot formation. Our study found that an ECOG performance status ≥ 2 was associated with a 13-fold increased risk of VTE. This finding is supported by Farmakis et al., who reported that patients with an ECOG score of ≥2 had elevated risks of cancer-associated VTE, including a two-fold higher risk of VTE recurrence and major bleeding events [31]. Similarly, Covut et al. observed that, in an MM cohort, a greater proportion of VTE patients exhibited a poor ECOG status (≥2) at 33%, compared to only 16% among those without VTE [32].

Our study found that recent orthopaedic surgery is a significant independent risk factor for VTE, with a 22-fold increased risk. This finding is compatible with existing literature that highlighted the elevated VTE risk associated with major orthopaedic surgeries, particularly those involving prolonged immobilisation. Rogers et al. reported an 11.8% incidence of post-surgical VTE within 90 days following fractures, emphasising the need for vigilant monitoring in this patient population [33]. Among the ten patients in our cohort who underwent orthopaedic-related surgery, four developed VTE after total hip replacement. Of the four patients who developed VTE, two also experienced pneumonia after surgery, and one had a surgical site infection that prolonged the recovery period. During these events, there was leukocytosis, and raised inflammatory markers, such as c-reactive protein, were observed. The increased risk can be attributed to several factors commonly associated with orthopaedic procedures, including surgical complexity, immobilisation, and postoperative complications such as bleeding and infection. A meta-analysis by Wang et al. further supported that these factors contribute significantly to VTE events in patients with hip fractures [34].

Our MM patients who experienced VTE had a higher disease burden at diagnosis, evidenced by lower Hb levels, reduced serum albumin, elevated β2-microglobulin, hypercalcemia, and increased lactate dehydrogenase (LDH) levels. These markers correspond with advanced disease stages (DS Stage III and Revised-ISS Stage III). However, these MM disease characteristics did not reach statistical significance in the univariate logistic regression analysis. These findings must be interpreted cautiously, as our sample size may have influenced these results. Early interventions, such as erythropoietin-stimulating agents to address anaemia, bisphosphonates for calcium management, and nutritional support, may improve patient outcomes and mobility by reducing infection risk, potentially lowering VTE risk, similar to effects seen in the general population [35].

Interestingly, a SAVED score ≥ 2 and IMPEDE-VTE score > 3 points were not significantly associated with VTE in the multivariate analysis. This finding may reflect differences in patient populations or variations in the predictive accuracy of the SAVED and IMPEDE-VTE models in specific settings. Similarly, treatment-related factors, such as thalidomide and autologous HSCT, did not reach statistical significance, possibly due to sample size limitations or confounding effects. In our study, we observed that two patients with venous thromboembolism (VTE) experienced bleeding complications while on rivaroxaban. In contrast, no bleeding events were reported among those treated with apixaban or enoxaparin. This highlights the variability in safety profiles among anticoagulants, particularly in MM treatment. The overall mortality rate in the VTE group was 22.7% (5 out of 22), with all deaths occurring within six months after the VTE events. Although Kristinsson et al. reported that MM patients with VTE have a 2.9-fold higher mortality rate (95% CI, 2.4–3.5) than those without VTE [36], our cohort was relatively small, making it difficult to draw firm conclusions. Additional research is required to better understand mortality rates and survival outcomes after VTE events in myeloma patients.

One notable strength of this study is its inclusion of NDMM patients from two high-volume tertiary centres, ensuring a comprehensive dataset that reflects Malaysia’s ethnically diverse population. Nonetheless, several limitations must be acknowledged. The retrospective nature of the study and the use of convenience sampling may have introduced a selection bias. Furthermore, confounding variables, such as the duration and cumulative doses of IMiDs or other novel agents, were not accounted for, potentially influencing the VTE incidence. The ability to compare VTE risk across specific treatment regimens was also limited due to frequent overlaps and crossovers in therapies based on the disease responses during the study period. Larger prospective studies are needed to validate our findings and clarify the multifactorial nature of VTE risk in MM patients.

## 5. Conclusions

In conclusion, our study’s incidence of VTE in NDMM patients was 10.2%, notably higher than the rates reported in other Asian cohorts. Key independent risk factors for VTE identified in our population included the female sex, an ECOG performance status of ≥2, and a recent orthopaedic surgery within six months of MM diagnosis. Developing an optimal thromboprophylaxis strategy for MM patients remains complex due to the concurrent risk of bleeding. Future research is essential to establish the most effective prophylactic agents and its duration, including the potential role of direct oral anticoagulants (DOACs) while accounting for their interactions with novel myeloma therapies.

## Figures and Tables

**Table 1:** Baseline characteristics of the study population.

Demographic Characteristic	All Patients (n = 216)	No VTE (n = 194)	VTE (n = 22)	*p*-Value
Host Factors				
Age, years, mean ± SD	62.4 ± 10.6	62.1 ± 10.7	64.5 ± 10.0	
<60	84 (38.9)	78 (92.9)	6 (7.1)	0.499 ^a^
60–70	82 (38.0)	72 (87.8)	10 (12.2)	
>70	50 (23.1)	44 (88.0)	6 (12.0)	
Sex				
Male	110 (50.9)	107 (97.3)	3 (2.7)	<0.001 ^a^
Female	106 (49.1)	87 (82.1)	19 (17.9)	
BMI, kg/m^2^				
Underweight, <18.5	52 (24.0)	45 (86.5)	7 (13.5)	0.0726 ^a^
Normal, 18.5–24.9	136 (63.0)	130 (95.6)	6 (4.4)	
Overweight and obese, ≥25.0	28 (13.0)	19 (67.9)	9 (3.2)	
Ethnicity				
Malay	130 (60.2)	114 (87.7)	16 (12.3)	0.085 ^b^
Chinese	58 (26.9)	52 (86.7)	6 (10.3)	
Indian	24 (11.1)	24 (100.0)	2 (0.0)	
Others	4 (1.9)	4 (100.0)	0 (0.0)	
Performance status, ECOG				
0	111 (51.4)	105 (94.6)	6 (5.4)	<0.001 ^b^
1	67 (31.0)	63 (94.0)	4 (6.0)	
2	33 (15.3)	23 (69.7)	10 (30.3)	
3	5 (2.3)	3 (60.0)	2 (40.0)	
Comorbidities				
Diabetes mellitus	154 (71.3)	134 (87.0)	20 (13.0)	0.032 ^a^
Chronic kidney disease	130 (60.2)	116 (89.2)	14 (10.8)	0.445 ^a^
Hypertension	90 (41.7)	85 (94.4)	5 (15.6)	0.207 ^a^
Dyslipidemia	18 (8.2)	14 (77.8)	4 (22.2)	0.094 ^b^
Ischemic heart disease	15 (6.8)	15 (100.0)	0 (0.0)	0.374 ^b^
Stroke	10 (4.5)	10 (100.0)	0 (0.0)	0.604 ^b^
Recent orthopaedic surgery < 6 months	10 (4.5)	6 (60.0)	4 (40.0)	0.011 ^b^
Disease Factors				
Type of MM				
Ig G kappa	81 (37.5)	72 (88.9)	9 (11.1)	0.390 ^b^
Ig G lambda	49 (22.7)	41 (83.7)	8 (16.3)	
Ig A kappa	32 (14.8)	30 (61.2)	2 (38.8)	
Ig A lambda	14 (6.5)	13 (92.9)	1 (7.1)	
Ig D kappa	1 (0.5)	1 (100.0)	0 (0.0)	
Kappa light chain	23 (10.6)	21 (91.3)	2 (8.7)	
Lambda light chain	16 (7.4)	16 (100.0)	0 (0.0)	
Disease Stages				
Durie–Salmon				
I	21 (9.7)	20 (95.2)	1 (4.8)	0.077 ^b^
II	38 (17.6)	37 (97.4)	1 (2.6)	
III	157 (58.8)	137 (87.3)	20 (12.7)	
R-ISS				
I	24 (11.1)	23 (95.8)	1 (4.2)	0.512 ^a^
II	45 (20.8)	41 (91.1)	4 (8.9)	
III	147 (68.1)	130 (88.4)	17 (11.6)	
Laboratory parameters at diagnosis				
Hb, g/dL, mean ± SD	8.31 ± 1.2	8.4 ± 1.2	7.2 ± 1.9	<0.001 ^c^
Β2-microglobulin, mg/L, median (IQR)	6.5 (5.7)	6.1 (5.6)	11.4 (6.2)	0.001 ^d^
Calcium, mmol/L, median (IQR)	3.30 (0.6)	3.30 (0.6)	3.40 (0.3)	<0.001 ^d^
Albumin, g/dL, median (IQR)	29.0 (3.0)	29.0 (3.0)	26.5 (8.5)	0.001 ^d^
LDH, U/L, median (IQR)	300.0 (110.0)	300.0 (102.5)	400.4 (142.5)	0.001 ^d^
Treatment Factors				
MM treatment in 1 year of diagnosis				
Thalidomide	179 (82.9)	160 (89.4)	19 (10.6)	0.646 ^a^
Lenalidomide	35 (16.2)	29 (82.9)	6 (17.1)	0.137 ^a^
Pomalidomide	16 (6.2)	15 (93.8)	1 (6.3)	0.498 ^b^
Bortezomib	162 (75.0)	149 (92.0)	13 (8.0)	0.069 ^a^
Cyclophosphamide	102 (47.2)	93 (91.2)	9 (8.8)	0.531 ^a^
Melphalan	21 (9.7)	18 (85.7)	3 (14.3)	0.363 ^b^
Autologous SCT	85 (39.4)	77 (90.6)	8 (9.4)	0.762 ^a^
Supportive treatment				
Radiotherapy	11 (5.1)	11 (5.1)	1 (9.1)	0.689 ^b^
CVC insertion	98 (45.4)	85 (86.7)	13 (13.3)	0.173 ^a^
Erythropoietin-stimulating agent	137 (63.4)	121 (88.3)	16 (11.7)	0.339 ^a^

Data are shown as n (%). SD standard deviation; IQR Interquartile range; ^a^: Chi-Square test; ^b^: Fisher’s Exact test; ^c^: Independent *t*-test; ^d^: Mann-Whitney test. Hb haemoglobin; LDH lactate Dehydrogenase; R-ISS Revised International Staging System; Autologous SCT Autologous stem cell transplants; CVC Central venous catheter.

**Table 2:** Risk assessment scores and VTE events.

	All Patients (n = 216)	No VTE (n = 190)	VTE (n = 22)	*p*-Value
SAVED Score				
Low Risk (−3–1 point)	206 (95.4)	190 (92.2)	16 (7.8)	<0.001 ^a^
High Risk (2–9 points)	10 (4.6)	4 (40.0)	6 (60.0)	
IMPEDE-VTE Score				
Low Risk (≤3 points)	149 (70.0)	142 (95.3)	7 (4.7)	0.001 ^a^
Intermediate Risk (4–7 points)	50 (23.1)	42 (84.0)	8 (16.0)	
High Risk (≥8 points)	17 (7.9)	10 (58.8)	7 (41.2)	

^a^ Chi-square test; VTE Venous thromboembolism.

**Table 3:** Details of the VTE events.

Parameter	n (%)
Time from myeloma diagnosis to VTE event	
0–1 month	2 (9.1)
1–6 months	14 (63.6)
6–12 months	6 (27.3)
Site of VTE	
DVT	15 (68.2)
Iliac vein	1
Femoral vein	9
Popliteal vein	4
Peroneal vein	1
PE	5 (22.7)
PE + DVT	2 (9.1)
Anticoagulant	
Direct oral anticoagulant	12 (54.5)
Rivaroxaban	8
Apixaban	4
Enoxaparin	10 (45.5)

DVT deep vein thrombosis; PE pulmonary embolism.

**Table 4:** Risk factors analysis for VTE in MM patients.

Variables	Univariate		Multivariate ^a^	
	Unadjusted OR (95% CI)	*p*-Value	Adjusted OR (95% CI)	*p*-Value
Female sex	7.79 (2.23, 27.19)	**0.001**	8.56 (1.95, 37.48)	**0.004**
ECOG PS				
Good (0–1)	1			
Poor (2–4)	7.75 (3.04, 19.76)	**<0.001**	12.74 (3.37, 48.17)	**<0.001**
Malay ethnicity	1.87 (0.70, 4.99)	0.210		-
Body mass index, kg/m^2^				
Not overweight (<25)	1			
Overweight and obese (≥25)	0.82 (0.97, 2.18)	0.248	-	
Diabetes mellitus	4.48 (1.01, 19.77)	**0.048**	3.24 (0.46, 22.91)	0.240
Chronic kidney disease	0.42 (0.17, 1.03)	0.057	-	-
Hypertension	1.62 (0.64, 4.08)	0.306	-	-
Dyslipidemia	2.86 (0.85, 9.60)	0.090	-	-
Recent orthopaedic surgery < 6 months	6.96 (1.78, 26.98)	**0.005**	21.79 (3.10, 153.38)	**0.002**
Stages of Disease				
Durie–Salmon I–II	1			
III	4.16 (0.94, 18.39)	0.060	-	-
Risk assessment model				
SAVED score				
<2 points	1			
≥2 points	17.81 (4.55, 69.70)	**<0.021**	18.55 (0.76, 452.36)	0.073
IMPEDE-VTE score				
≤3 points	1			
>3 points	8.59 (2.86, 25.80)	**<0.040**	3.07 (0.18, 51.97)	0.438
Treatment Factor				
Autologous SCT	0.87 (0.35, 2.17)	0.762	-	-
Thalidomide	1.35 (0.38, 4.80)	0.647	-	-
Lenalidomide	2.13 (0.77, 5.90)	0.145	-	-
Cyclophosphamide	0.75 (0.31, 1.84)	0.532	-	-
Melphalan	1.54 (0.42, 5.72)	0.516	-	-

^a^ Adjusted for female sex, poor ECOG status, recent orthopaedic surgery, diabetes mellitus, SAVED score > 2, and IMPEDE-VTE score > 3; OR, odds ratio; CI, confidence interval; ECOG PS, Eastern Cooperative Oncology Group (ECOG) performance status; Autologous SCT, autologous stem cell transplants; (-) not proceeded in multivariate analysis; and *p*-values < 0.05 are shown in bold, indicating significance.

## Data Availability

The original contributions presented in the study are included in the article material. Further inquiries can be directed to the corresponding author.
